# A Mixed-Method Evaluation of a Prison Anti-doping Intervention: The Hercules Prison Program

**DOI:** 10.3389/fspor.2021.779218

**Published:** 2021-12-23

**Authors:** Dominic Sagoe, Berit Johnsen, Bo Lindblad, Tom Are Jensen Normann, Vidar Skogvoll, Morten Heierdal, Fredrik Lauritzen

**Affiliations:** ^1^Department of Psychosocial Science, University of Bergen, Bergen, Norway; ^2^University College of Norwegian Correctional Service (Kriminalomsorgens Høgskole og Utdanningssenter KRUS), Lillestrøm, Norway; ^3^Department of Prevention and Public Health, Anti-doping Norway, Oslo, Norway; ^4^Science and Medicine, Anti-doping Norway, Oslo, Norway

**Keywords:** anabolic steroids, anti-doping, Hercules program, prevention, prison, strength training

## Abstract

The Norwegian Offender Mental Health and Addiction study denotes the need for physical activity and anti-doping interventions in Norwegian prisons. We developed and evaluated the efficacy of such intervention—the Hercules prison program. The program combines theoretical anti-doping lessons with practical strength training. The study adopts a mixed-methods approach (pretest-posttest design) comprising a longitudinal survey, observation, informal conversations, and in-depth interviews. Survey respondents were 104 male prisoners aged 18–56 (*M* = 34.81, *SD* = 9.34) years from seven Norwegian prisons. Of these, 52 provided both baseline and posttest responses. Participants completed questionnaires including demographic, doping use, and psychophysical items/measures. At the end of the intervention, in-depth interviews were conducted with 11 of the survey respondents. The survey data were analyzed using descriptive statistics, as well as independent and paired samples *t*-tests. The qualitative data were analyzed using Interpretative Phenomenological Analysis. A total of 7.5% and 33.3% of participants were current and former AAS users respectively, whereas 86.1% personally knew at least one current or former AAS user. Consistent with our expectation, there were increases in self-rated physical strength (*t* = −4.1, *p* < 0.001, *d* = 0.46) and strength training self-efficacy (*t* = −8.33, *p* < 0.001, *d* = 1.36), and a decrease in moral disengagement in doping (*t* = −4.05, *p* < 0.001, *d* = 0.52) from baseline to posttest. These findings are supported by the qualitative data. Notable success factors are relationship-building, instructors' expertise and acceptability, and gatekeepers' navigation and co-creation. The program provides valuable evidence of the potential benefits of combining anti-doping education with practical strength training in doping prevention in correctional settings.

## Introduction

There is a high prevalence of drug use among prisoners with overall lifetime prevalence estimates of 30% in male prisoners and 51% in female prisoners (Fazel et al., 2017). Particularly, the lifetime prevalence of anabolic-androgenic steroid (AAS) use among prisoners and arrestees is 12.4% (Sagoe et al., 2014). In the Nordic countries, prisoners and arrestees constitute a major subgroup of AAS users with a lifetime prevalence of 26.2% (Sagoe et al., 2015a). Analysis of data from 1,499 prisoners participating in the Norwegian Offender Mental Health and Addiction (NorMA) study (Bukten et al., 2016) shows that 65% had used drugs for intoxication, and 35% during previous or current prison stays. Twenty-three percent had used AAS which was more prevalent among men (25%) than women (4%). Further analysis of data from 1,464 prisoners from the NorMA study shows that lifetime prevalence of drug and unprescribed medication, mostly Image and Performance Enhancing Drugs (IPED), use during incarceration was 23.1%, with 1.6% reporting lifetime use of AAS during incarceration (Muller et al., 2018).

Physical activity has been shown to improve substance use treatment outcomes in terms of increased abstinence and decreased relapse, enhanced physical strength and well-being, improved self-esteem and health awareness, decreased depression (Williams and Strean, 2004) as well as enhanced relaxation and mood (Fitzgerald et al., 2021). The prison setting is ideal for physical activity interventions as there is an absence of important barriers to physical activity such as cost, insufficient time, partner and family commitment and issues, absence of equipment, and transportation to training facilities (Plugge et al., 2011; Parker et al., 2014; Jones et al., 2021). Physical activity interventions among prisoners have been associated with increased physical health and strength, higher self-esteem and self-efficacy, improved mood and reduced aggressive behavior, and enhanced mental health (Mohan et al., 2018; Psychou et al., 2019, 2020).

Norwegian prisons, as other prisons in the Nordic countries, are renowned for being humane and prioritizing rehabilitation (Pratt, 2008; Pratt and Eriksson, 2014; Andvig et al., 2020). Moreover, substance use treatment is an integral part of prisoner rehabilitation and rehabilitative interventions, particularly on substance use, are encouraged in Norwegian prisons (Giertsen, 2012; Helgesen, 2015, 2019; Mjåland, 2016). As evidence from the NorMA study shows high prevalences of substance use and lack of physical activity among Norwegian prisoners (Bukten et al., 2016; Muller et al., 2018), there is a need for interventions that address both substance use and lack of physical activity in this group (Muller et al., 2018). With the proliferation of the use of IPEDs such AAS into the general population (Sagoe et al., 2014; Sagoe and Pallesen, 2018), anti-doping interventions are recommended for non-sports settings (Bates et al., 2019). Particularly, there is a need for anti-doping interventions for prisoners with a history of or who currently use IPEDs such as AAS. We have previously (Sagoe et al., 2016) implemented and evaluated the efficacy of a doping prevention program for adolescents/high school students—the Hercules program. The Hercules program for adolescents (Sagoe et al., 2016) demonstrates the benefits of combining anti-doping education with practical strength training in doping prevention.

Against this backdrop, the present project implemented and evaluated the effectiveness of an anti-doping intervention for Norwegian prisoners (Hercules Prison Program) using a mixed-method approach. From the standpoint of the integrative model of behavioral prediction (Fishbein, 2000, 2008), doping use is influenced by intention, with skills and environmental constraints moderating the link between doping use and intention. As a precursor to doping use, intention is influenced by attitude and perceived norm and self-efficacy. From a quantitative viewpoint, it is important that the responses of participants are examined statistically. Additionally, from a qualitative perspective, an exploration of the lived experiences of participants and program implementers is elucidating (Parker et al., 2014).

The project has two main aims. First, we examine the effectiveness of the Hercules prison program as a prison-based anti-doping intervention. Second, we explore participants' and program implementers' experiences of the Hercules program. The overarching research question guiding the project is: is the Hercules prison program an effective prison-based anti-doping intervention? From a quantitative viewpoint, we reasonably expected an increase in physical strength from baseline to posttest. Additionally, we expected (Ntoumanis et al., 2014; Sagoe et al., 2016) increases in protective factors (strength training self-efficacy, ability to turn down drug offers, and muscle appearance satisfaction) and decreases in risk factors (AAS use intent and moral disengagement in doping) of doping from baseline to posttest. The key research question guiding the qualitative part is: what are participants' and program implementers' experiences of the Hercules program?

## Methods

### Design

We used a mixed methods approach comprising a pretest-posttest design. Data were collected using a longitudinal survey (quantitative), in-depth interviews, informal conversations, and observation (qualitative). The qualitative part of the study followed a fieldwork approach. Fieldwork is social in its essence (Hammersley and Atkinson, 2019). The researchers take part in the social world that is to be studied and the data is produced in interaction with the respondents. Building rapport with the respondents is essential to conducting successful fieldwork (Bernard, 2017), and prior understandings and knowledge of the field is vital for this process. Prior understandings also influence what knowledge is produced in the field. Whereas the in-depth interviews were useful for exploring participants' experiences, the informal conversations were useful for engendering rapport, trust and understanding, and the observation for discerning participants' behavior during the intervention. Additionally, the study implemented a two-phase sequential explanatory design with the qualitative data building on and elucidating the quantitative data (Ivankova et al., 2006).

### Participants and Procedure

**Figure 1:**
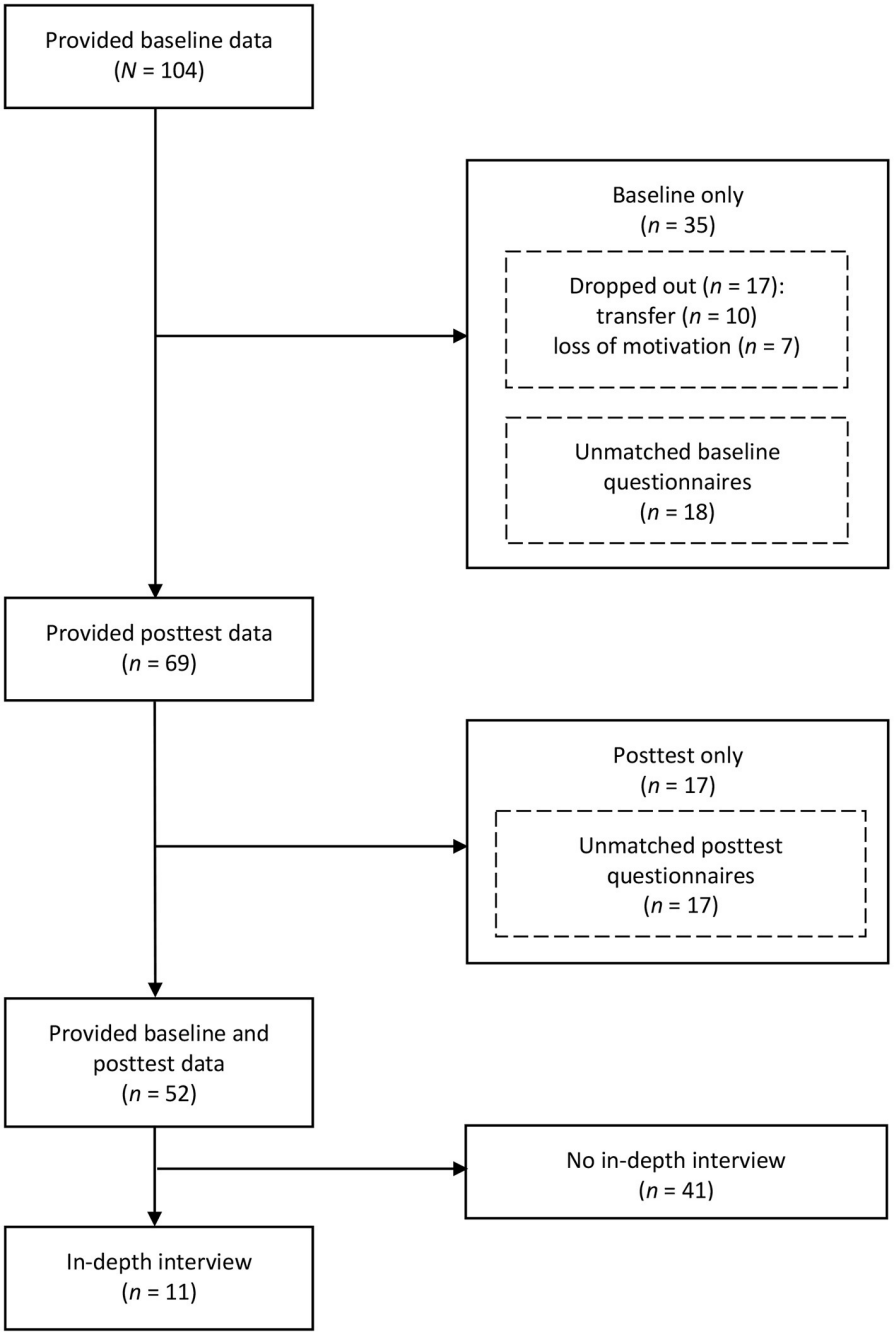
Study flow chart presenting number of respondents at different assessment points.

Survey respondents were 104 male prisoners aged 18 to 56 (*M* = 34.81, *SD* = 9.34) years from seven prisons (Berg, Eidsberg, Halden, Ila, Ringerike, Skien, and Ullersmo) in Norway. Of these, 52 aged 19–52 (*M* = 34.14, *SD* = 9.18) years returned matched questionnaires to both the baseline and posttest measures. Dropout from the study was due to being transferred to another prison or ward (*n* = 10) or loss of motivation (*n* = 7). There were 35 unmatched questionnaires. To avoid false matching and duplication while enhancing inclusivity, the 35 unmatched questionnaires were scrutinized and sorted by respondents' prison. Next, 18 questionnaires from the baseline assessment were added to the 17 dropouts (baseline only; *n* = 35) and 17 from the posttest were coded as posttest only. Excluding the 10 transferred baseline participants and the 17 unmatched posttest questionnaires, the attrition rate was 32.47% (25/77). Eleven participants from two units were recruited during the intervention for the in-depth interviews using convenience sampling. The study flow chart indicating number of participants at different assessment points is presented in Figure 1, and participant characteristics in Table 1.

**Table 1:** Characteristics of the study sample (*N* = 104).

**Variable**	***n***	**%**
Sample (*N* = 104)		
Baseline–posttest	52	50.00
Baseline only	35	33.65
Posttest only	17	16.35
AAS use§ (*n* = 93)		
Currently	7	7.53
Past (“lifetime”)	31	33.33
Never	55	59.14
Know AAS user(s)§ (*n* = 86)		
No	12	13.95
Yes	74	86.05
	**Range**	***M*** **(*****SD*****)**
Age, y (*n* = 101)§	18–56	34.81 (9.34)
Self-rated physical strength (*n* = 85)^‡^	1–5	3.42 (0.92)
AAS use intent (*n* = 73)^‡^	5–24	10.11 (4.80)
Strength training self-efficacy (*n* = 83)^‡^	7–30	19.53 (4.40)
Ability to turn down drug offers (*n* = 84)^‡^	4–20	16.69 (4.03)
Muscle appearance satisfaction (*n* = 85)^‡^	7–25	16.45 (4.46)
Moral disengagement in doping (*n* = 84)^‡^	6–23	13.39 (4.31)

§ *Baseline (± posttest)*.

‡ *Baseline only*.

Participants were recruited from addiction treatment units of the prisons through convenience sampling. The intervention is a good fit for prisoners in these units as they follow a treatment program that provides stability for the intervention. Prisoners in these units also have group sessions and have their meals together making it easier for recruitment and the arrangement of the theoretical part of the intervention. Two of the authors (VS and TAJN) who are prison officers and are familiar with the prison setting followed-up the intervention in two departments. At the first meeting, the prisoners were informed of their background and affiliation to the Norwegian Correctional Service. Respondents then signed the consent form to participate in the study. Observation and informal conversations were carried out during the teaching and practical strength training sessions provided by two instructors (BL, MH) while they trained with participants. Here, BL's expertise in exercise science and coaching, and MH's familiarity with the prison setting as well as their roles as instructors facilitated their fieldwork. Also, VS and TAJN's knowledge and familiarity of the prison setting before being participating observers during the intervention was important.

In-depth face-to-face interviews were conducted by VS or TAJN at the end of the intervention. The interviews lasted from 30 to 75 min and all, but one, were taped and later transcribed by the interviewer. Notes were taken throughout the interview that was not taped, and transcribed immediately after the interview. Concerning the observation, one department was visited nine times and the other eight. The visits lasted between two and three hours. The quantitative data collection was conducted in the autumns of 2019 and 2020, and the qualitative data in autumn 2019. We conducted the study in line with the declaration of Helsinki and ethical approval was obtained from the Norwegian Regional Committee for Medical and Health Research Ethics—North (2019/1142/REK nord). We present the study according to the Guidelines for Conducting and Reporting Mixed Research in the Field of Counseling and Beyond (Leech and Onwuegbuzie, 2010).

### Program Description

**Figure 2:**
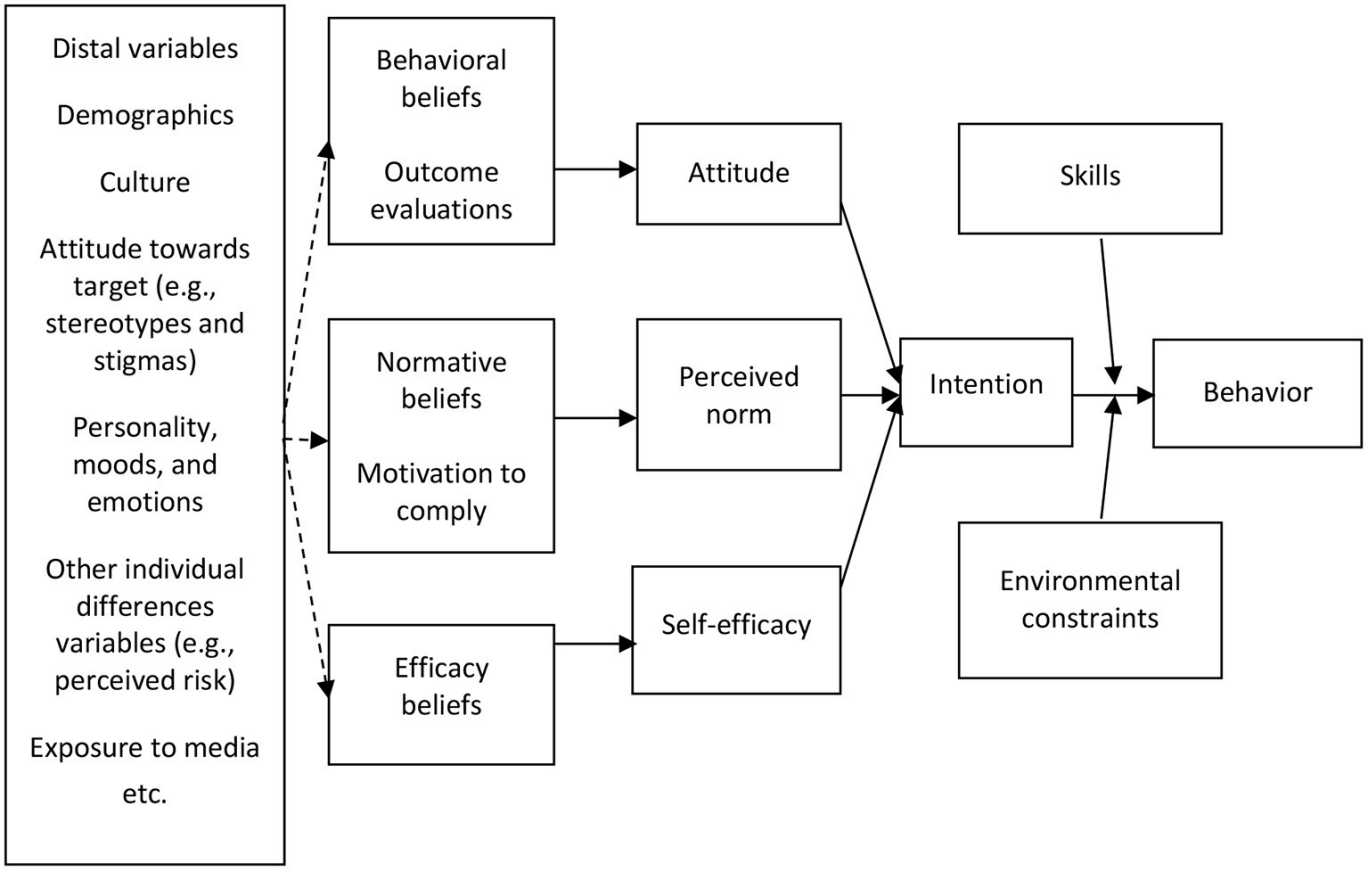
The integrative model of behavioral prediction (Fishbein, 2000, 2008).

The program is anchored in the integrative model of behavioral prediction (Fishbein, 2000, 2008) presented in Figure 2. We preferred this model as it is the theoretical foundation for the preponderance of research on doping use and anti-doping (Ntoumanis et al., 2014). From this model, doping use is influenced by intention (e.g., AAS use intent), with skills (e.g., ability to turn down drug offers) and environmental constraints (e.g., doping milieu) moderating the link between doping use and intention. As a precursor to doping use, intention is influenced by attitude and perceived norm (e.g., moral disengagement in doping), and self-efficacy (e.g., strength training self-efficacy). Thus, the above factors were incorporated in the program and are accordingly assessed using a self-report questionnaire.

The program lasted nine consecutive weeks and is based on previous work by our group with adolescents–the Hercules program for adolescents (Sagoe et al., 2016). The intervention comprised four 3–4-h personalized sessions including a combination of relationship building, theoretical lectures, discussions, and practical strength training. During each visit, the two program instructors (BL and MH), had time to talk individually with participants and shared a meal at the end of the session. An overview of the program is presented in Table 2.

**Table 2:** Overview of the Hercules prison program.

**Session**	**Description**
Week 1, visit 1	Introduction to the positive and negative effects of doping use with emphasis on AAS. A program instructor shared a personal experience of doping use and incarceration
Week 2, visit 2	Theoretical lectures on strength training theory, exercise planning, sport nutrition and the effects and risks of dietary supplements. The session ended with a practical exercise session where participants were introduced to an introductory strength training program and proper lifting technique
Weeks 3 and 4	Three strength training bouts per week under supervision
Week 5, visit 3	Introduction to a new exercise program with emphasis on maximum strength training. Instructors and participants went through all the new exercises together during which each participant received personal feedback and guidance
Weeks 6–8	Three strength training bouts per week under supervision
Week 9, visit 4	Instructors repeated the theoretical lectures from week 1 in a shortened version and participants were provided an advanced training strength training program to stimulate further physical activity. The session ended with a joint reflection meeting on participants' lived experiences of program participation. They also received an Anti-Doping Norway branded water bottle as a token of appreciation for their participation in the program

### Measures

#### Quantitative

All participants completed a paper questionnaire consisting of the following variables.

##### Age

Participants indicated their ages in years.

##### Self-Rated Physical Strength

Self-rated physical strength was assessed with the question: “How would you describe your physical strength?” Response options were: 1 (far below average), 2 (slightly below average), 3 (average), 4 (slightly above average), and 5 (far above average)—“compared with my peers.” Self-rated physical strength has been assessed using similar questions in previous studies (Mikkelsson et al., 2005; Petersen et al., 2021; Sjøgaard et al., 2021).

##### AAS Use

We assessed AAS use with the question: “Have you ever used anabolic-androgenic steroids?” with response options being 0 (no), 1 (yes, but no more), and 2 (yes and currently). To verify AAS use, participants indicated the AAS they had used by answering the question: “If yes, what anabolic steroids or similar substances have you used?” Participants also indicated whether they personally knew a current or former AAS user (yes/no).

##### AAS Use Intent

AAS use intent was assessed with the Intent to Use AAS Scale (IAS; MacKinnon et al., 2001). The IAS comprises five items such as “I am curious to try anabolic steroids.” Each item is rated on a 5-point Likert scale ranging from 1 (strongly disagree) to 5 (strongly agree). Total scores range between 1 and 25 with higher scores signifying higher intent to use AAS. For the present study, the internal consistency assessed by Cronbach's alpha were 0.95 at baseline and 0.90 at posttest.

##### Strength Training Self-Efficacy

Efficacy at strength training was assessed using the Strength Training Self-Efficacy Scale (STSES; MacKinnon et al., 2001): The STSES consists of six items such as “I know how to train with weights to increase my endurance.” Each item is answered on a 5-point Likert scale ranging from 1 (strongly disagree) to 5 (strongly agree). Accordingly, total scores range between 6 and 30 with higher scores denoting higher strength training efficacy. Cronbach's alphas of the STSES were 0.82 and 0.78 at baseline and posttest respectively.

##### Ability to Turn Down Drug Offers

Ability to turn down offers to use AAS and other drugs was assessed using the Ability to Say No to Drugs Scale (ASNDS; MacKinnon et al., 2001). The ASNDS contains four items (e.g., “I would be comfortable turning down a weightlifter who offered me anabolic steroids”) with each item answered on a Likert scale ranging from 1 (strongly disagree) to 5 (strongly agree). Total scores range from 4 to 20 with higher scores indicating higher ability to reject drug offers. Cronbach's alphas were 0.89 at baseline and 0.84 at posttest.

##### Muscle Appearance Satisfaction

We used a short version of the Muscle Appearance Satisfaction Scale (MASS; Ryan and Morrison, 2010) in assessing participants' satisfaction with the appearance of their muscles as well as muscle dysmorphia symptoms. MASS contains 6 items (e.g., “I often spend money on muscle-building supplements”) answered on a Likert scale ranging from (1) strongly disagree to (5) strongly agree. MASS yields a total composite score ranging between 6 and 30 with higher scores signifying higher satisfaction with one's muscle appearance and lower muscle dysmorphia symptoms. At baseline, MASS yielded a Cronbach's alpha of 0.75, and 0.82 at posttest.

##### Moral Disengagement in Doping

We used the Moral Disengagement in Doping Scale (MDDS; Kavussanu et al., 2016) in assessing participants' moral disengagement in doping. The MDDS consists of six items such as: “Doping does not really hurt anyone.” Each item is rated on a Likert scale ranging from 1 (strongly disagree) to 7 (strongly agree). The MDDS total scores range from 6 to 42 with higher scores indicating higher moral disengagement in doping. Cronbach's alpha values were 0.75 at baseline and 0.67 at posttest.

#### Qualitative

The qualitative part of the study consisted of observation, informal conversations with participants during the observational sessions, and in-depth interviews. The interviewees were recruited during the intervention and the interviews were carried out at the end of the intervention. Participants' experiences of the intervention were gathered using an adapted version of an interview guide (Dowrick et al., 2013). Aspects of the interview guide and example questions are provided in Table 3.

**Table 3:** Overview of the semi-structured interview guide.

**Aspect**	**Example questions**
Discovery	How did you first find out about the program? Who told you about it?
Engagement	What made you decide to try the program? (Try to find out about background/personal circumstances of participant)
Participation	Did the facilitator persuade you to try something that you might not have thought of?
Experiences	Who led the sessions? What do you think of their approach?
Outcomes	Do you think that taking part in the program has had an effect on you personally (positive/negative)? Has it helped you? In what ways?

### Data Analysis

For the quantitative study, we used descriptive statistics in terms of frequencies and proportions as well as means and standard deviations to ascertain characteristics of the sample. Additionally, we conducted an independent *t*-test to compare characteristics of lifetime AAS users and non-users at baseline for the overall sample as well as for the baseline-posttest participants. Finally, based on a modified intention-to-treat (mITT) approach, we tested for the efficacy of the intervention by conducting a paired samples *t*-test to compare participants' scores at baseline and at posttest. Here, *post-hoc* power analysis setting *d* to 0.5, alpha to 0.05, and 52 participants indicated a power of 0.98.

The qualitative data were analyzed using Interpretative Phenomenological Analysis (Smith et al., 1995). The analysis of the qualitative data was conducted in two phases. The first phase had a “bottom-up” approach where nine central themes (the intervention; drugs and narcotics; health; capital; diet; body; life; methods; exercise) were identified as codes under which the data were categorized. In the second phase, six of the nine codes (the intervention; drugs and narcotics; health; capital; diet; body) were re-coded in accordance with the variables in the survey (self-rated physical strength; AAS use intent; strength training self-efficacy; ability to turn down drug offers; muscle appearance satisfaction; moral disengagement in doping). Also, based on participants' experiences from the six codes, three success factors were identified: relationship-building, instructors' expertise and acceptability, and gatekeepers' navigation and co-creation. Thus, in addition to the themes, the statistical findings and participants' perspectives are further explored under the three success factors. The statistical analysis was conducted using SPSS version 25 (IBM Corp.) and the power analysis using G^*^Power 3 (Faul et al., 2009). The qualitative analysis was conducted using NVivo (QSR International).

## Results and Discussion

We first present findings on AAS us prevalence and compare AAS users and non-users on survey measures. Next, we discuss statistically significant findings and elaborate them with participants' experiences and field notes followed by a delineation of statistically non-significant findings. We then discuss intervention success factors. The implications of findings are also described, and the section ends with a consideration of the strengths and limitations of the study, as well as recommendations for future research.

### AAS Use Prevalence

A total of 7.5% and 33.3% of participants indicated being current and former AAS users respectively. Additionally, 86.1% of participants indicated personally knowing at least one current or former AAS user (see Table 1). Several participants described their experiences of using AAS, including the positive effects such as increased confidence and strength (Bonnecaze et al., 2020) as well as the harms such as depression (Griffiths et al., 2018), rage and aggression (Chegeni et al., 2021; Pope et al., 2021), inflammation and gynecomastia (Albano et al., 2021). One participant indicated that:

I exercised twice a day, and used steroids the whole week, and the results were extremely good. I have always been so thin … It [steroids] was good for me in many ways. I increased my self-confidence and such things, but it also had negative effects. I will not only talk about the positive things about doping, it brings about a lot of shit too (Participant quote 2).

The estimated lifetime prevalence of AAS use in the present study is consistent with a previous meta-analytic estimate among prisoners and offenders in the Nordic countries (Sagoe et al., 2015), as well as recent NorMA estimates (Bukten et al., 2016; Muller et al., 2018; Havnes et al., 2020). Juxtaposed with the current prevalence estimate, as well as the estimate of knowing a current or former AAS user, it is inferable that a large majority of prisoners are exposed to AAS-using or “dopogenic” (Backhouse et al., 2018) milieu/social networks such as acquaintances, family members, teammates, and gymnasiums that facilitate or reinforce doping use. A more detailed discussion of this finding is presented in a previous publication (Johnsen et al., 2021).

### AAS Users vs. Non-users

Results of the between-group comparison (“lifetime” AAS users vs. non-users) for the overall and the baseline-posttest samples are presented in Tables 4, 5 respectively. For the overall sample, “lifetime” AAS-using participants had higher (*t* = −2.32, *p* < 0.05, Cohen's *d* = 0.54) self-rated physical strength than the non-using participants. This result was however not replicated in the baseline-posttest participants (*t* = −1.39, *p* = 0.209, Cohen's *d* = 0.42). Additionally, “lifetime” AAS-using participants had higher AAS use intent compared to the non-using participants in both the overall (−2.85, *p* < 0.01, Cohen's *d* = 0.73) and baseline-posttest (*t* = −3.33, *p* < 0.01, Cohen's *d* = 1.07) samples. Moreover, “lifetime” AAS-using participants had higher moral disengagement in doping compared to the non-using participants in the overall (*t* = −3.13, *p* < 0.01, Cohen's *d* = 0.72) as well as the baseline-posttest (*t* = −2.83, *p* < 0.01, Cohen's *d* = 0.85) samples.

**Table 4:** Baseline comparison of “lifetime” AAS-using (AAS+) and non-using (AAS–) prisoners for the overall sample.

**Variable**		**AAS+**	**AAS–**	
	**Range**	***M* (*SD*)**	***M* (*SD*)**	***t***
Age (AAS+: *n* = 31; AAS–: *n* = 54)	18–56	36.29 (9.56)	33.75 (9.18)	−1.20
Self-rated physical strength (AAS+: *n* = 30; AAS–: *n* = 54)	1–5	3.73 (0.74)	3.26 (0.98)	–2.32^*^
AAS use intent (AAS+: *n* = 19; AAS–: *n* = 54)	5–24	12.68 (5.12)	9.20 (4.38)	–2.85^**^
Strength training self-efficacy (AAS+: *n* = 30; AAS–: *n* = 52)	7–30	20.70 (3.50)	18.81 (4.77)	−1.90
Ability to turn down drug offers (AAS+: *n* = 30; AAS–: *n* = 54)	4–20	17.03 (3.55)	16.50 (4.30)	−0.58
Muscle appearance satisfaction (AAS+: *n* = 31; AAS–: *n* = 53)	7–25	17.55 (4.31)	15.85 (4.50)	−1.70
Moral disengagement in doping (AAS+: *n* = 31; AAS–: *n* = 53)	6–23	15.23 (3.79)	12.32 (4.27)	–3.13^**^

** *p < 0.01*,

* *p < 0.05*.

**Table 5:** Baseline comparison of baseline-posttest “lifetime” AAS-users (AAS+) and non-users (AAS–).

**Variable**		**AAS+ (*n* = 18)**	**AAS– (*n* = 34)**	
	**Range**	***M* (*SD*)**	***M* (*SD*)**	***t***
Age	19–56	35.78 (9.63)	33.24 (8.96)	−0.94
Self-rated physical strength	1–5	3.72 (0.75)	3.35 (0.98)	−1.39
AAS use intent	5–24	14.92 (5.92)	9.68 (4.37)	–3.33^*^
Strength training self-efficacy	9–26	21.00 (2.91)	18.88 (4.14)	−1.92
Ability to turn down drug offers	4–20	16.94 (3.17)	16.64 (4.08)	−0.27
Muscle appearance satisfaction	8–26	17.94 (5.20)	16.81 (4.22)	−0.84
Moral disengagement in doping	6–23	15.72 (3.64)	12.31 (4.31)	–2.83^*^

* *p < 0.01*.

Our finding in the overall sample that “lifetime” AAS users had higher self-rated physical strength than non-using participants may reflect the strength benefits of AAS (Andrews et al., 2018). Also, our finding that “lifetime” AAS users had higher AAS use intent and moral disengagement in doping compared to non-users is in line with the integrative model of behavioral prediction (Fishbein, 2000, 2008). Here, it is reasonable that the elevated levels of AAS use intent and moral disengagement in doping in “lifetime” AAS users are influenced by positive attitudes, normalization, and self-efficacy (Sagoe, 2014) from previous exposure to AAS and “dopogenic” environments (Backhouse et al., 2018).

### Intervention Outcomes and Experiences

#### Self-Rated Physical Strength and Strength Training Self-Efficacy

Results of the statistical comparison of participants' baseline-posttest scores on self-rated physical strength and strength training self-efficacy are presented in Table 6.

**Table 6:** Comparison of baseline-posttest participants' (*n* = 52) scores.

**Variable**	**Baseline**		**Posttest**		**Comparison**		
	**Range**	***M* (*SD*)**	**Range**	***M* (*SD*)**	***t***	***p***	***d***
Self-rated physical strength	1–5	3.41 (0.91)	2–5	3.78 (0.69)	−4.05	**0.000**	0.46
AAS use intent	5–23	9.44 (4.88)	5–19	8.17 (3.92)	1.72	0.094	4.43
Strength training self-efficacy	7–30	19.28 (4.78)	17–30	24.67 (2.96)	−8.33	**0.000**	1.36
Ability to turn down drug offers	4–20	16.88 (3.73)	4–20	17.26 (3.25)	−0.57	0.571	0.11
Muscle appearance satisfaction	7–26	16.08 (4.58)	6–25	16.80 (4.96)	−1.21	0.232	0.15
Moral disengagement in doping	6–22	13.50 (4.07)	6–20	11.56 (3.38)	−4.05	**0.000**	0.52

Consistent with our hypothesis, participants reported an overall increase (*t* = −4.1, *p* < 0.001, Cohen's *d* = 0.46) in self-rated physical strength from baseline (*M* = 3.41, *SD* = 0.91) to posttest (*M* = 3.78, *SD* = 0.69). There was also an increase in strength training self-efficacy (*t* = −8.33, *p* < 0.001, Cohen's *d* = 1.36) from baseline (*M* = 19.28, *SD* = 4.78) to posttest (*M* = 24.67, *SD* = 2.96) in line with our prediction. These findings are in line with evidence from the previous Hercules program for adolescents (Sagoe et al., 2016) as well as from previous physical activity interventions in prisons (Mohan et al., 2018; Sanchez-Lastra et al., 2019; Papa et al., 2021).

Participants' experiences of enhanced physical strength (self-rated) and improved strength training self-efficacy support the above statistical findings. All participants had previous strength training experience. Their knowledge about training had mostly been acquired from their peers and based on so-called “bro science” (Havnes and Skogheim, 2019; Harvey et al., 2020) or folk pharmacology (Southgate and Hopwood, 2001; Underwood, 2017) which is strongly influenced by a “macho” or “hardcore” culture characterized by excessive training and heavy lifts with large muscles as the only measure of success (Midgley et al., 2001; Denham, 2008; Turnock, 2021). This approach to strength training had afflicted many participants with musculoskeletal pain and injuries, and subsequent physical inactivity prior to the intervention. However, the instructors motivated and guided these participants to exercise safely by personalizing the program as recommended (Brighton et al., 2020; Harvey et al., 2020). This was appreciated:

[The participant] came over to me and said that he liked it [the program] very much. He had not been exercising for years… he has been afraid of exercising because of an injured knee. However, he felt safe when the instructor gave him recommendations of how he could exercise despite the injury. [Another participant] was given alternative exercises because of his back pain (Field note 1).

One participant with a previous injury indicated that: “they [the instructors] dissuade you from the bad things and show you the right way, I think” (Participant quote 1). Many participants indicated experiencing an improvement in their training techniques: “I have improved my techniques—that is how I lift the weights, how I grab them, my position, heights and regulation of the levels” (Participant quote 1). These participants felt they could cope with the program and had rediscovered the joy of physical exercise. Experienced weightlifters also benefitted from the individual guidance such as when they sought direction from instructors on proper technique for specific exercises. The program represented a new way of exercise for many: “Earlier, we lifted as much as we could on the bench press until we had no energy left. Now [with this program], the exercise is more varied and we get tired in a new manner” (Participant quote 2). Although the program had a rigid structure, with the help of the instructors, some participants made adjustments to the program so it better fitted their needs. Some supplemented weight training with exercises such as football and running. Many adapted the program to their daily routines.

In addition to the practical knowledge acquired, participants also found the theoretical knowledge from the lessons useful. At the beginning of the intervention, some participants were skeptical about the nutritional advice given by the instructors. Some were particularly skeptical of the scientific advice on protein needs and recommended intake provided during the lectures. This skeptical attitude is not surprising (Pope et al., 2004; Zahnow et al., 2017; Bonnecaze et al., 2020). However, during the program, many gradually changed their opinion and indicated that they would change their diet regimen. One indicated: “I have been drinking a lot of protein shakes, but I will not do that anymore. The clue is to have a varied diet” (Participant quote 3). Some participants also explained learning the importance of rest and not putting too much strain on their bodies in order to obtain good results from the training. As one participant expressed:

I like people who say that you don't need to put on weights as hell. You should actually not strain your body as much as you think. I used to think “the heavier, the better,” but it isn't like that at all. Rest is important (Participant quote 3).

Although participants of the qualitative study in unison appreciated the intervention, their answer to the question about following the program after completing the intervention was more ambiguous. While some indicated that they would continue, others said that they would incorporate some of the exercises in their future training programs.

#### Moral Disengagement in Doping

Moral disengagement in doping refers to the facilitation of doping use through personal considerations such as utilitarianism (e.g., doping hurts nobody but helps the team), normalization (e.g., doping is normal in sports), rationalization (e.g., doping maximizes potential), and loss of control, e.g., people cannot be blamed for doping if their teammates are doping (Lucidi et al., 2008; Kavussanu et al., 2016). There was decreased moral disengagement in doping (*t* = −4.05, *p* < 0.001, Cohen's *d* = 0.52) from baseline (*M* = 13.50, *SD* = 4.07) to posttest (*M* = 11.56, *SD* = 3.38) in line with our expectation (see Table 6).

The first teaching session, where one of the instructors shared his personal experience of AAS use and the side effects, had a great impact on participants: “[The instructor's] history opened something in the group by touching something very personal in each of them” (Field note 2). During the intervention, this instructor was available and obliging for individual talks and guidance, which the prisoners appreciated very much. Although the emotional aspect was not very apparent in the lessons where doping was discussed, this aspect reflected in the social atmosphere of the intervention. At the concluding and debriefing session, a similar atmosphere was observed when the side effects of doping use were discussed. While some participants thought that the instructors exaggerated the side effects during the first teaching session, there was unanimous agreement during the last session that the instructors had not exaggerated the side effects.

The instructors' non-judgmental attitude and recognizing rather than undermining the positive effects of AAS use also generated trustworthiness in the participants (Goldberg et al., 1991; Bates, 2019; Ainsworth, 2020). One participant opined: “Such scaremongering, it doesn't work. It's better to do it in the way they [the instructors] have. Yes, it's [AAS] bloody good, it works as hell, but it has also negative effects” (Participant quote 2).

#### AAS Use Intent

In contrast to our hypothesis, we did not observe a difference in (*t* = 1.7, *p* = 0.094, Cohen's *d* = 4.43) in AAS use intent from baseline to posttest. However, many of the participants with AAS use experience indicated ambivalence to the use of AAS. One reason for this may be that several of them have not only used AAS to increase their performance but they have also used AAS in combination with other drugs consistent with evidence in the field (Sagoe et al., 2015b; Havnes et al., 2020; Smit et al., 2020; Piatkowski et al., 2021). Despite their ambivalence of using AAS themselves, most participants indicated that they would not recommend this to others: “I would not recommend steroids to my son, if I had one” (Participant quote 5). Another participant indicated:

After this intervention I have got more respect for steroids. I'm more conscious that I will not use it again. Earlier, I was a bit unsure, and I didn't know much about it before they [the instructors] arrived. I had a chat with [one of the instructors], and I learned what this actually does to you. This door is now closed (Participant quote 4).

The interviews show that AAS non-using participants have been offered AAS through their association with drug milieus, but they had turned such offers down as they were afraid of side effects, especially after having witnessed side effects in others: “Where I come from, there are many users. I have met people that have been using [AAS] and they are unstable: grown up men that cry or yell. I have understood that it has done something to their heads” (Participant quote 3).

#### Ability to Turn Down Drug Offers and Muscle Appearance Satisfaction

We did not observe differences in the ability to turn down drug offers (*t* = −0.6, *p* = 0.571, Cohen's *d* = 0.11) and muscle appearance satisfaction (*t* = −1.2, *p* = 0.232, Cohen's *d* = 0.15) from baseline to posttest in contrast to our expectation. An inspection of the interview transcripts and field notes did not reveal intervention-relevant experiences on these two factors.

### Intervention Success Factors

The above statistical findings and participants' perspectives on enhanced physical strength (self-rated), improved strength training self-efficacy, and decreased moral disengagement in doping underline the potential of the Hercules prison program as an effective prison-based anti-doping intervention. This potential can be further highlighted under the three success factors: relationship-building, instructors' expertise and acceptability, and gatekeepers' navigation and co-creation.

#### Relationship-Building

Relationship-building is a notable success point of the intervention in line with evidence from a recent meta-synthesis indicating the importance of relationship-building in exercise-based interventions (Brighton et al., 2020). There were high expectations in several of the prison groups. A department head pointed out that: “The inmates were excited and ready to receive us. They had had a countdown and were looking forward to this meeting [with Anti-Doping Norway]” (Field note 1). The experience of joining the meal sessions of participants during the introductory session was positive. The arena around the table was “neutral” and instructors and participants met as “equal” parties who could freely share their experiences about doping, physical training and other issues. Thus, began the relationship-building process.

The first teaching session was a catalyst for goal achievement for the intervention. The introduction with presentation and exchange of experiences was relatable to participants. An instructor's narration of his lived experience was important here: “[One of the instructors] presented himself and his story as a former AAS user and inmate. This worked very well, and it was clear that participants were touched by the story and recognized themselves in [his story]” (Field note 1). This is a crucial success factor as improved communication and relationships has been found to facilitate program participation and improve outcomes (Brighton et al., 2020). Thus, in addition to the teaching sessions, there were many forms of informal interaction and exchanges during the breaks and lunch which facilitated relationship building. Additionally, Anti-Doping Norway staff's supervision of and participation in the strength training sessions as well as their motivation of and follow-up of participants was of great value to the intervention.

#### Instructors' Expertise and Acceptability

From a holistic approach, evidence from a systematic meta-synthesis based on the experiences of both participants and program implementers suggests that the expertise of program implementers is crucial for their acceptability and program success (Brighton et al., 2020). Beyond relationship building, the two instructors (BL, MH) from Anti-Doping Norway have a rich expertise and experience that the participants appreciated. As indicated previously, BL has a college degree in exercise science and coaching whereas MH has a long experience of health club environments, is a former prisoner and has a personal experience of AAS use reported widely in Norwegian media. We noted this in the field notes: “[The instructors] had a good interaction with participants, where one described the [AAS] user perspective while [the other] supported scientifically” (Field note 1). As noted previously, the instructors' background made them more acceptable to the participating inmates.

During the first teaching session, the transition from lived experience to the science of exercise, health, nutrition, and diet on another instructor's part generated initial skepticism in some participants. This initial skepticism gradually disappeared during the practical strength training session. One participant described this: “It's good to get it [exercise guidance] from professionals. Then, I trust it more” (Participant quote 6). The teaching sessions and individual guidance during the practical strength training sessions also created a safe space and empowered participants to learn and freely participate in conversations about strength training, doping and other substance use. The individual guidance also involved a recognition of the participants' efforts and training skills: “I liked that [the instructor] gave me a thumbs up when I did the exercises. Got some recognition from someone who knew this stuff (Participant quote 5).

#### Gatekeepers' Navigation and Co-creation

In qualitative research, gatekeepers are persons or institutions possessing the power and authority to grant or deny the researcher access to participants or the research setting (Andoh-Arthur, 2019). Also, co-creation refers to collaboration between scientists, clinicians, policymakers and other stakeholders or gatekeepers in knowledge generation (Greenhalgh et al., 2016). It is notable that the key hindrance to the success of a previous intervention was the inability to navigate institutional gatekeeping (Gil-Delgado et al., 2011). In the present project, the Norwegian Correctional Service was a key gatekeeper as their permission was indispensable in accessing the prison setting. In this regard, their scientific collaboration as evident in authorship (BJ, TAJN, VS) facilitated access to permission and program success. In a co-creation paradigm (Ramaswamy and Gouillart, 2010), actively involving participants in physical activity intervention design in prison settings may lead to benefits such as participant empowerment, intervention acceptability, and enhanced trust and communication (Greenhalgh et al., 2016; Mohan et al., 2018).

In navigating ethical obstacles with actively involving prisoners in the design of our intervention (Goodyear-Smith et al., 2015), we relied on the experience of a former inmate (MH), presently an anti-doping campaigner, as indicated previously. However, some participants served as gatekeepers in the intervention implementation due to their respected status in the hierarchical structure of prison society (Ugelvik, 2014; Kreager et al., 2017). They motivated and positively influenced the exercise regimen of the other participants. Based on recommendation from an influential prisoner, prisoners in one department resorted to drinking a tuna shake—a self-composed protein shake made from canned tuna and used by several of the inmates—to maximize the effect of strength training. During the intervention, participants understood that this was not necessary: “I'm done with drinking tuna shakes. A glass of milk is enough…and traditional food” (Participant quote 7). In general, the recognition of the skills of participants with previous strength training experience was important:

I liked the approach from you [the instructors]. Not something from top to bottom. There was a lot of knowledge with them, but no one reprimanded us and said that what we had previously learnt about various exercises or that what we could do [prior to the intervention] was wrong (Participant quote 3).

The gatekeepers' navigation and co-creation approach, specifically the collaboration between a national anti-doping organization (Anti-Doping Norway) and correctional service (Norwegian Correctional Service), and academia (University College of Norwegian Correctional Service, and University of Bergen), is endorsed by the European Union's Experts in Doping Prevention in Recreational Sports (Backhouse et al., 2014) and other anti-doping (Gatterer et al., 2020) and correctional experts (Dumont et al., 2021).

### Implications of Findings

As noted previously, our prevalence estimates point to the exposure of many prisoners to AAS-using or “dopogenic” milieu (Backhouse et al., 2018) and underline the need for milieu therapy (Giannini et al., 1991; Bruhn et al., 2017; Terry et al., 2021) for prevention, harm reduction, and treatment in this population. Based on the integrative model of behavioral prediction (Fishbein, 2000, 2008), it is important that, under milieu therapy, consideration is given to dealing with the positive attitudes, normalization, and self-efficacy (Sagoe, 2014) as well as the “bro science” (Havnes and Skogheim, 2019; Harvey et al., 2020) that reinforce AAS and other IPED use. Additionally, the statistical results and participants' experiences underline the anti-doping potential of the Hercules prison program. As such, the Hercules prison program has potential as a novel anti-doping intervention for correctional settings. Replication of the program in other jurisdictional prisons must take into consideration the exceptionality of Norway's prison setting in prioritizing rehabilitation and humane treatment of prisoners (Pratt and Eriksson, 2014; Johnsen and Fridhov, 2018; Dugdale and Hean, 2021). Moreover, such interventions must consider the success points of relationship-building, instructors' expertise and acceptability, and incorporation of gatekeepers previously elucidated.

### Strengths, Limitations, and Directions for Future Research

To our knowledge, the Hercules prison program is the first anti-doping intervention in a correctional setting (Bates et al., 2019). The potential benefits of the program is underlined in the medium effect size for self-rated physical strength and the large effect sizes for both strength training self-efficacy and moral disengagement in doping (Cohen, 1988). Relatedly, although the pretest-posttest difference for AAS use intent, ability to turn down drug offers, and muscle appearance satisfaction did not reach statistical significance, they changed in the direction of our hypotheses. Conceivably, the intervention success factors of relationship-building, instructors' expertise and acceptability, and gatekeepers are generic strengths of the study. Specifically, the mixed-method design presenting the advantages of quantification and exploration of participants' lived experiences is a key strength of the study. Here, the use of well-validated instruments is a strength particularly of the quantitative aspect of the study. The combination of theoretical lessons and practical strength training is another strength of the intervention.

However, data were collected using self-reports which may have limited validity especially in prison settings where responses are sometimes characterized by social desirability due to perceived consequences (Mielitz and MacDonald, 2020; Sivakumar, 2021). In particular, self-ratings of physical strength are relative (“compared with my peers”) rather than absolute and are therefore probably affected by rank order fluctuation, especially considering the hierarchical structure of prison society (Ugelvik, 2014; Kreager et al., 2017) as noted previously. Also, although the sample size appears low, it is justified by power analysis and is equivalent to the preponderance of samples from similar interventions on this topic (Mohan et al., 2018; Legrand et al., 2020; Pralong et al., 2020; Psychou et al., 2020; Papa et al., 2021).

A key limitation is our within-subjects design. In contrast to the Hercules program for adolescents (Sagoe et al., 2016), it was not pragmatic (in terms of sample recruitment) to include control and theory only groups as well as female prisoners. Our results on the baseline-posttest comparison of participants may therefore reflect history, maturation or testing effects rather than intervention effects. Accordingly, control and theory only groups and female prisoners should be considered in future studies for improved analysis of intervention outcomes. Additionally, our recruitment of participants from addiction treatment units of the prisons through convenience sampling may reflect selection bias. Although the posttest Cronbach's alpha for MDDS was relatively low, it is higher than the 0.60 cut-off score recommended for short scales (Loewenthal, 2001) and therefore acceptable.

Moreover, the ability to turn down drug offers (as a resistance skill; MacKinnon et al., 2001) and moral disengagement in doping assessed in our study may arguably not aptly represent (actual) skills and perceived norm respectively in terms of the integrative model of behavioral prediction (Fishbein, 2000, 2008). A longer intervention including post-incarceration follow-up may also elucidate the effect of the intervention on actual behavior change (anti-doping behavior) and make an incremental contribution to this line of research. Furthermore, similar scientific and policy collaboration between national anti-doping organizations, correctional services, and academia is encouraged for the advancement of anti-doping, sports, and correctional science. From a co-creation perspective (Ramaswamy and Gouillart, 2010), future interventions may benefit from the active involvement of participants in intervention design (Greenhalgh et al., 2016; Mohan et al., 2018).

## Conclusion

Our statistical findings and participants' perspectives on enhanced physical strength (self-rated), improved strength training self-efficacy, and decreased moral disengagement in doping point to the potential of the Hercules prison program as an anti-doping intervention in correctional settings. The program highlights the potential benefits of combining anti-doping education with practical strength training in doping prevention in correctional settings.

## Data Availability Statement

The raw data supporting the conclusions of this article will be made available by the authors, without undue reservation.

## Ethics Statement

Ethical approval was obtained from the Norwegian Regional Committee for Medical and Health Research Ethics—North (2019/1142/REK nord). The patients/participants provided their written informed consent to participate in this study.

## Author Contributions

BL, MH, and FL designed the intervention in consultation with DS. DS obtained ethical approval for the intervention. BL and MH were instructors. BL, TN, VS, MH, and FL collected the data. DS, BJ, VS, and TN conducted the data coding, transcription, and analysis. All authors contributed to the study design, writing and revision process, and approved the final manuscript.

## Funding

The Norwegian Directorate of Health through a grant to Anti-Doping Norway funded the present study.

## Conflict of Interest

The authors declare that the research was conducted in the absence of any commercial or financial relationships that could be construed as a potential conflict of interest.

## Publisher's Note

All claims expressed in this article are solely those of the authors and do not necessarily represent those of their affiliated organizations, or those of the publisher, the editors and the reviewers. Any product that may be evaluated in this article, or claim that may be made by its manufacturer, is not guaranteed or endorsed by the publisher.
